# Three-dimensional in vitro culture models in oncology research

**DOI:** 10.1186/s13578-022-00887-3

**Published:** 2022-09-11

**Authors:** Camille Jubelin, Javier Muñoz-Garcia, Laurent Griscom, Denis Cochonneau, Emilie Ollivier, Marie-Françoise Heymann, François M. Vallette, Lisa Oliver, Dominique Heymann

**Affiliations:** 1 Nantes Université, CNRS, US2B, UMR 6286, 44000 Nantes, France; 2 Institut de Cancérologie de l’Ouest, Tumor Heterogeneity and Precision Medicine Lab.. Université de Nantes, 44805 Saint-Herblain, France; 3 Atlantic Bone Screen, Saint-Herblain, France; 4 Univ Rennes, CNRS, INSERM, Biosit UAR 3480 US_S 018, BIM3D core facility, Rennes, France; 5 Nantes Université, INSERM, CRCINA, UMR1232, Nantes, France; 6 CHU de Nantes, Nantes, France; 7 Department of Oncology and Metabolism, Medical School, University of Sheffield, Sheffield, UK

**Keywords:** 3D cell culture, Multicellular tumor spheroid, Organoid, Liquid-based 3D culture, Scaffold-based 3D culture, Microfluidics, Bioprinting, Cancer

## Abstract

Cancer is a multifactorial disease that is responsible for 10 million deaths per year. The intra- and inter-heterogeneity of malignant tumors make it difficult to develop single targeted approaches. Similarly, their diversity requires various models to investigate the mechanisms involved in cancer initiation, progression, drug resistance and recurrence. Of the in vitro cell-based models, monolayer adherent (also known as 2D culture) cell cultures have been used for the longest time. However, it appears that they are often less appropriate than the three-dimensional (3D) cell culture approach for mimicking the biological behavior of tumor cells, in particular the mechanisms leading to therapeutic escape and drug resistance. Multicellular tumor spheroids are widely used to study cancers in 3D, and can be generated by a multiplicity of techniques, such as liquid-based and scaffold-based 3D cultures, microfluidics and bioprinting. Organoids are more complex 3D models than multicellular tumor spheroids because they are generated from stem cells isolated from patients and are considered as powerful tools to reproduce the disease development in vitro. The present review provides an overview of the various 3D culture models that have been set up to study cancer development and drug response. The advantages of 3D models compared to 2D cell cultures, the limitations, and the fields of application of these models and their techniques of production are also discussed.

## Introduction

With around 10 million deaths per year, cancer is one of the main causes of mortality in industrial countries after cardiovascular diseases and is consequently a major public health problem [1]. The decline in overall cancer mortality observed over the last two decades can be attributed more to better prevention and early detection methods than to breakthroughs in treatment even though significant pharmacological and therapeutic progress has been made [2]. However, these successes vary greatly depending on the type of cancer [3]. Cancer is a multifactorial disease arising from cells that adopt abnormal behaviors, including sustained proliferative molecular networks (e.g. drug resistance, cell death resistance, angiogenesis) and immune evasion properties leading to the replicative immortality and invasion/migration characteristics associated with metastases [4].

Malignant tumors are most often considered as heterogeneous tissues including inter- and intra-tumor heterogeneity [5, 6]. Inter-tumor heterogeneity refers to the composition and organization diversity observed for a given type of cancer between patients. Intra-tumor heterogeneity corresponds to the intrinsic cellular and molecular heterogeneity within tumor tissue. Intra-tumor heterogeneity does not only refer to the clonal diversity of cancer cells, but also to the heterogeneity of the tumor microenvironment [7]. Both are frequently amplified by selective pressures, and particularly by therapies that lead to the acquisition of drug resistance profiles in the cancer cells responsible for therapeutic failure [8, 9]. Intra-tumor heterogeneity can be considered a dynamic process (e.g. modification of genetic features through selection mechanisms, as well as morphological and phenotypical changes over time and space) that impose continual therapeutic adaptation.

To investigate the mechanisms involved in cancer initiation, progression and drug resistance, diverse models have been proposed. In vivo models (e.g. mice, rats, dogs, monkeys, pig) are based on spontaneous or induced malignant tumors (e.g. inoculation of cancer cells). Spontaneous models consist of de novo generation of cancer, either using genetically engineered animal models (GEMs) that reproduce germline mutations observed in patients [10], or subjecting animals to radiation, virus or chemical carcinogens [11, 12]. The spontaneous models have the advantage of mimicking all the steps in tumor growth in immunocompetent mice, however, the tumors generated are the result of oncogenic events that may be quite different from the natural history of cancer cells. In addition, the tumor microenvironment remains of murine origin, which may limit some therapeutic developments. Allografts or xenografts are faster methods for generating tumors in vivo. However, the frequency of tumor development may be lower, and once again the local microenvironment is murine [13]. In vivo experiments make it possible to investigate cancer in a highly complex microenvironment similar but not identical to that observed in patients, but their drawbacks include complex analyses, major ethical concerns, and such experiments are time- and resource-consuming requiring trained staff.

In vitro cultures entail growing cells derived from multicellular organisms in plastic or glass culture dishes. In vitro cultures have the advantage of being highly controllable, making possible easily repeatable experiments, being mostly inexpensive, and leading to a vast range of applications. The most widely used in vitro technique is two-dimensional (2D) cell culture, where cells grow as adherent monolayers on culture vessels. However, 2D cultures remain over-simplified models that do not mimic tissue organization in vivo, and in particular the tumor microenvironment (TME). The TME is composed of cellular components, such as immune cells (T cells, B cells, Natural killer cells, macrophages, neutrophils, dendritic cells) and stromal cells (endothelial cells, cancer-associated fibroblasts (CAFs), adipocytes), and of non-cellular components such as the extracellular matrix (ECM) which is a network of macromolecules (mainly proteoglycans, collagen, laminin, elastin, fibronectin, and enzymes), water, cytokines and growth factors [14–16]. The composition and proportion of each component of the TME depend on the tumor host tissue, the type of cancer and the patient [17–19]. The TME has been shown to play a major role in tumorigenesis, cancer progression and cancer resistance, and should be taken into account when studying cancer in vitro. The enrichment of 2D cell cultures with other cell types from the TME and/or non-cellular components improves the 2D culture models by partially reproducing the in vivo microenvironment. But 2D cell cultures cannot depict the dimensional organization of a complete tumor mass. Three-dimensional (3D) culture models have the potential to bridge the gap between 2D in vitro models and in vivo models. The present review aims to describe the main 3D culture models currently available for studying cancer development and drug screening in oncology, and to discuss their added value and specific limitations.

## In vitro cancer modelling: 2D or not 2D?

Cancer cells cultured in 2D do not have the same morphology, heterogeneous phenotype, extracellular matrix (ECM) or gene and protein expression profile as cells cultured in 3D.

### 2D cell cultures do not depict the biological realities of tumors

After tissue cultures were established by Harrison in the early twentieth century [20], mammalian cell cultures developed rapidly with various culture dishes with or without treated surfaces, different cell culture media, and the creation of cell repositories around the world. Because 2D adherent monolayer cell cultures (in temperature, hygrometry and CO_2_ controlled environment) produce reproducible results and are easy to implement, inexpensive, and easy to analyze, they have become a widely used tool in biological studies and as well as being the standard for in vitro cancer research. Admittedly, 2D cultures have contributed tremendously to expand knowledge of cancer; however, it has become obvious that these simplistic models cannot depict the biological reality of tumors and their TME.

All the limitations of 2D cultures are inherently linked to the proliferation of the cells as an adherent monolayer on a plastic surface, as opposed to the 3D arrangement observed in vivo, and the morphology is the main differential feature between the culture modes. In 2D, adherent cells spread on the surface of the culture dishes with a flattened morphology. These cells have the ability to translate the mechanical forces of their environment into biochemical signals through mechano-transduction [21]. It has been shown in noncancerous cell lines that the cells can detect the stiffness of the substrate to which they attach [22], leading to cytoskeleton remodeling [23], differential proliferation and cell death [24]. In the case of chondrocytes, their spreading when cultured as adherent monolayers was associated with the initiation of a dedifferentiated phenotype and cytoskeletal changes in comparison to the in vivo and 3D cell culture phenotype [25, 26].

In addition, the physical organization of the monolayer greatly limits cell-to-cell interactions and prevents the formation of a transport gradient. In 2D cultures, cells have equal and unlimited access to the nutrients and their response to molecular cues such as chemotherapy is different from the in vivo situation. Furthermore, the spatial and molecular composition of the ECM produced by cancer cells is strongly affected by the culture method [27]. The ECM plays a crucial role in tumorigenesis, tumor progression, and migration by modulating signaling events through contact and growth factors binding to cell-surface receptors [16, 28–33]. In 3D cultures and in vivo*,* cells are present as multilayers and as such are not exposed to the same concentration of oxygen, nutrients and signaling molecules depending on their distance from blood vessels. Various physiological processes are based on this transport gradient, i.e. proliferation and angiogenesis. In this context, to stay close to the in vivo tumor organization, pseudo-3D cell cultures (also called 2.5D cell cultures) were developed, such as growing cells as an adherent monolayer on ECM-coated culture vessels [34], using micropatterned platforms [35] or culturing cancer cell spheroids on top of a cell-sheet [36]. However, these pseudo-3D cell cultures do not efficiently replicate tumors and their microenvironment, because all the cells are still attached to their substrate on one side with the other side exposed to the liquid media.

### Taking a step further in mimicking the disease with spheroids and organoids

Spheroids are generated either by self-assembling cancer cell lines in suspension or dissociating patient tumor tissue. They are easy to produce and handle, and they are especially powerful for studying micrometastases or avascular tumors [37, 38]. It is possible to improve their relevance by integrating cells from the TME (e.g. CAFs, immune cells, vascular cells) into the spheroids [39, 40]. For all these reasons, since their first use in 1970 by Inch et al*.* [41], spheroids are the most used 3D model in cancer research.

However, spheroids remain simple models that only partially represent the in vivo organization and microenvironment of tumors. Organoids are more complex 3D models. Organoids are self-organized organotypic cultures that arise from tissue-specific adult stem cells (ASC), embryonic stem cells (ESC) or induced pluripotent stem cells (iPSC). ESC- or iPSC-derived organoids make it possible to generate the complex structure of adult organs in which all cells are fully differentiated. They may contain mesenchymal, epithelial, and even endothelial components [42]. However, these organoids tend to retain fetal properties and contain cells that should not be found in this type of tissue [43]. On the other hand, ASC-derived organoids are not as complex and can only be generated from adult tissues that retain regenerative properties, but they better reproduce adult tissues [44]. Although ESC- and iPSC-derived organoids are particularly useful for studying organogenesis and genetic pathologies, ASC-derived organoids have the advantage of mimicking both physiological and pathological adult tissues such as tumors. In 2009, Sato et al*.* developed a protocol for producing organoids of the intestinal epithelium by growing leucine-rich repeat-containing G protein-coupled receptor 5 positive (LGR5+) intestinal stem cells in medium containing stem cell niche-recapitulating and tissue-specific growth factors. At the same time, another organoid culture method was proposed by Ootani et al*.* [45]. Instead of growing isolated stem cells in a submerged manner, they used the Boyden chambers system. Tissue fragments containing both epithelial and stromal cells were embedded in a layer of ECM gels in direct contact with air. This layer sat on a porous membrane that allowed nutrients to diffuse from the bottom compartment containing medium. The advantage of this method over the submerged method is that it allows better oxygenation of the organoids and thus grows large multicellular organoids. Moreover, the stromal cells are enough to support organoid survival and no growth factor supplementation is required. In both culture methods, matrix is required to grow the organoids.

**Fig. 1:**
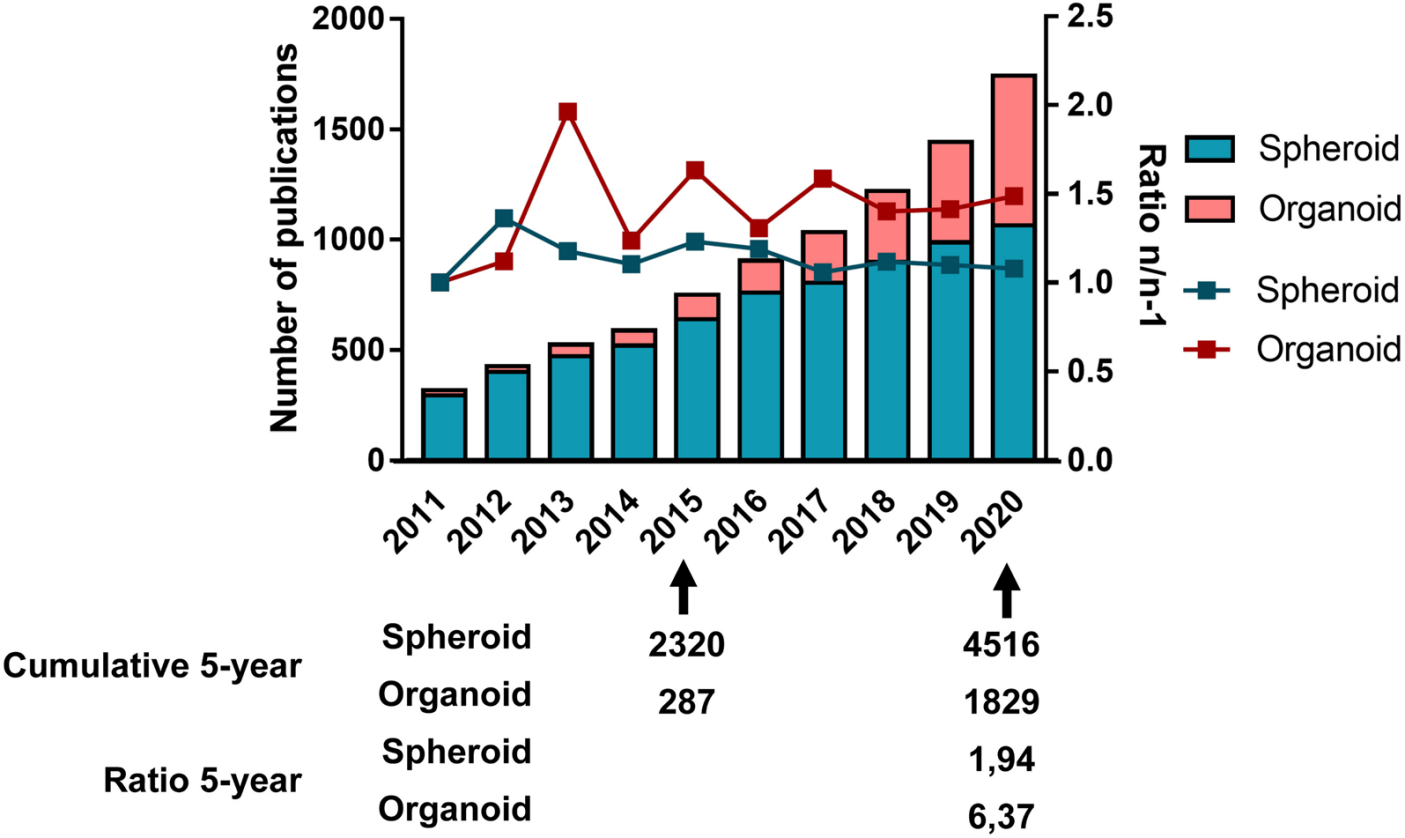
Comparison of the publication rate on spheroids and organoids over the past decade. For spheroids, the query used was: [(((Cancer) OR (Neoplasms)) AND ((spheroid) OR (tumorosphere))) NOT (Review)]. For organoids, the query used was: [(((Cancer) OR (Neoplasms)) AND ((organoid) OR (tumoroid))) NOT (Review)]

A search for primary articles in PUBMED concerning organoids used in cancer research with the query [(((Cancer) OR (Neoplasms)) AND ((organoid) OR (tumoroid))) NOT (Review)] yields the results of 261 publications on this theme between 2011 and 2015, and 1693 between 2016 and 2020. A similar search using the query [(((Cancer) OR (Neoplasms)) AND ((spheroid) OR (tumorosphere))) NOT (Review)] displays 2,078 publications about spheroids and cancer between 2011 and 2015, and 4,126 between 2016 and 2020. While spheroid research led to almost twice as many publications in the past 5 years compared to the first half of the decade, the publication rate on organoids increased more than six times in the same time interval (Fig. 1). This underlines the increasing interest of the scientific community in organoids as oncology research models.

### Why and how 3D can bridge the gap between in vitro and in vivo studies

A tumor mass is characterized by the coexistence of a heterogenic population of cancer cells in close contact with the ECM and a particular microenvironment. In this context, 3D cell cultures in the form of spheroids or organoids form a biological tool capable of replicating the cellular heterogeneity present in tumors—as opposed to the homogeneity observed in 2D cell cultures. Moreover, as the transport gradient of oxygen, nutrients and cellular waste is generally limited to 150–200 µm, spheroids or organoids with a diameter of more than 500 µm present a stratified structure with a proliferating cell population localized at their periphery and a core which is composed of non-dividing and necrotic cells [46]. This spherical organization is very similar to what is observed in vivo in avascular tumor masses and is related to drug sensitivity. In addition, 3D cell cultures at least partially reproduce the tumor microenvironment by restoring cell-to-cell and cell-to-ECM interaction. For instance, the cell adhesion molecule e-cadherin, which generates epithelial cell–cell binding and is also involved in cancer initiation and progression, was shown to be present in higher levels in epithelial breast carcinoma MCF-7 cells or colon adenocarcinoma Lovo cells when cultured in 3D, which also resulted in higher chemo-resistance to cisplatin, 5-fluorouracil and Adriamycin. When these cells were treated with an anti-e-cadherin neutralizing antibody, chemosensitivity was restored and the result was similar to that detected in 2D cultures [47]. Similarly, cell-to-ECM interactions are involved in drug resistance [48].

3D cultures greatly contributed to better understanding various aspects of cancer biology, such as tumor progression, tumor microenvironment, gene and protein expression, pro-oncogenic signaling pathways, and drug resistance. 3D cultures also seem to be a promising platform for drug developments and screenings, including immunotherapies.

#### Tumor progression

Tumor progression involves various processes, including carcinogenesis, angiogenesis and metastasis. For each of these mechanisms, the tumor microenvironment is a key player. Roulis et al*.* demonstrated the role of the microenvironment on the initiation of colorectal cancer (CRC) in mice and patients. Using organoids containing epithelial and mesenchymal cells, these authors showed that colorectal carcinogenesis of mutated stem cells is controlled by neighboring Ptsg2-expressing fibroblasts through paracrine signaling. These fibroblasts secrete prostaglandin E2 (PGE2) that dephosphorylates the oncoprotein Yes-associated protein (YAP) in the stem cells, leading to YAP nuclear translocation, where it can activate the genes involved in cell proliferation and inhibit apoptotic genes. Roulis et al*.* also observed that the culture of the organoids with PGE2 drives the formation of a spheroid-like structure that is associated with poor differentiation and increased stemness, whereas inhibition of the PGE2 receptor in stem cells allowed organoids to form budding organoids with crypt-villus architecture [49]. Vasculogenesis is a physiological process observed during organ development and regeneration by which endothelial cells vascularize tissues in an organotypic manner. It is also a process found in cancer. Palikuqi et al*.* transduced adult endothelial cells with the ETS Variant Transcription Factor 2(ETV2) gene, which is normally only expressed during vasculogenesis in embryos. These authors observed that ETV2 expression allowed endothelial cells to (re)acquire vasculogenic and adaptable properties. When grown in 3D in a matrix composed of a mixture of laminin, entactin and type-IV collagen (LEC matrix), this ETV-expressing endothelial cells were able to form stable vessels. The authors call them R-VEC for ‘reset’ vascular endothelial cells. Using a microfluidic device base in fibrin gel, R-VEC cells were able to grow into a sprouting vascular network that supported gravity-perfused transport of whole blood without losing its integrity. In in vivo experiments in mice, R-VEC cells injected into LEC matrix formed durable non-leaky vessels that were anastomosing to the mouse vasculature. Moreover, R-VEC co-cultured with organoids were able to arborize these organoids. Palikuqi et al*.* observed differences in the clustering pattern and gene expression of R-VECs when co-cultured with healthy colon-organoids (COs) or with patient-derived colorectal cancer (CRC) organoids. A signature typical of tumor endothelial cells was detected when R-VECs were co-cultured with CRC organoids. With this work, Palikuqi et al*.* established a model of vasculogenesis that could help study the vascular niches of tumors [50]. The metastatic process is a multi-step event that involves migration of cancer cells from the primary tumor site through vasculature, invasion into secondary sites and metastatic development. During migration, cancer cells have to make their way into a confining environment. By culturing single breast cancer cells into a hydrogel made of basement membrane and alginate with tunable plasticity, Wisdom et al*.* showed that cancer cells were able to deform the matrix through the mechanical action of their invadopodia and migrate into the pore they carved. When lowering the plasticity of the matrix, this protease-independent migration was reduced [51]. To study collective cell migration [52], which is another form of migration than the traditional single cell migration described in the metastatic process, Huang et al*.* co-cultured tumorigenic breast cancer cell lines and non-tumorigenic epithelial breast cell lines as spheroids that they embedded into a collagen matrix inside a microfluidic platform. They observed that the architecture of the spheroids varied over time and that this organization was responsible for different invasive properties, underlying the importance of 3D co-culture models for fully understanding the role of cell–cell interaction in the migration process [53].

#### Signaling pathways

Differences in cancer cell behavior from 3D compared to 2D cultures come from changes in signaling pathways. Shifts in the catabolism pathways used by cancer cells can be observed when cultured in 2D vs. 3D [54]. Transformed MCF10A H-Ras mammary epithelial cells and human and murine breast cancer cell lines consumed proline when grown in 3D, whereas they secreted proline in 2D. This proline catabolism helped 3D growth by sustaining ATP production. On the other hand, non-transformed mammary epithelial cells did not use this pathway during acini formation in 3D, implying that proline catabolism is specific to breast cancer cells cultured in 3D. Furthermore, proline catabolism is higher in metastases compared to primary breast cancers in patients and mice, and inhibition of this pathway impairs the formation of lung metastases in mice. Therefore, by growing cancer cells in 3D, Elia et al*.* discovered a potential drug target against metastasis formation in breast cancer [54]. The secretome profile of cancer cells is also different in 2D or 3D. Hela cervical cancer cells cultured in 3D secreted extracellular vesicles (EVs) with a miRNA profile much more similar (96%) to cervical cancer patients’ circulating EVs, compared to 2D-derived EVs. The culture condition, however, did not affect the DNA profile of the EVs. Taken together, these results suggest that 3D culture methods may be more relevant for studying the miRNA secretome profile of cancer cells [55]. Similarly, other signaling pathways involved in cancer are affected by the method of culture and can explain differences in treatment responses observed in traditional 2D cultures compared to in vivo*.* When cultured in 3D, various colon cancer cell lines displayed a reduced AKT/mTOR/S6K pathway compared to 2D cultures, which is a signaling pathway that is involved in carcinogenesis, cancer cell migration and resistance to treatments [56]. 3D cultures of ER + /Her2 + breast cancer cell lines promote switching of the AKT to MAPK pathway, leading to reduced sensitivity to treatments [57].

#### Gene and protein expression

The gradient of nutrients, oxygen and cellular waste, the hypoxic conditions, the cell-to-cell and cell-to-ECM interactions generated in 3D cultures are all molecular cues that affect cellular physiology and, in turn, alter the gene and protein expression that can be involved in drug resistance. Zschenker et al*.* observed differences in gene expression profiles between 2 and 3D cell cultures. When cultured in 3D, A549 lung tumor cells and UT-SCC15 squamous carcinoma cells showed better survival rates after radio- and chemotherapy as compared to 2D culture conditions. In addition, the cancer cells grown in 3D also displayed significant altered gene expression with regard to tissue development, cell adhesion and defense responses [58]. Changes in gene expression profiles that are involved in mechanisms such as autophagy, stemness, cell cycle, apoptosis, cell migration and invasion have also been described [59–62]. The microenvironment is also involved in differential gene expression. CRC organoids cultured alone lacked the gene expression involved in cell–cell communication with the microenvironment that were detected in the original tumor tissues. When the CRC organoids were co-cultured with CAFs, the organoids started to re-express in a patient-dependent manner various genes that were present in the original tumor tissue and that have been reported as holding oncogenic functions [63]. Moreover, spheroid and organoid models mimic the in vivo tumor [64–67]. Genetic analyses have shown that organoids established from biopsies of metastatic CRC patients were similar to their initial metastasis. Ninety percent of somatic mutations were shared between tumor biopsy and organoid cultures, and none of the mutations that are specific to the tumor or organoid model are found in driver genes or genes that could serve as a drug target [66]. The histological and cytological properties, markers and mutations observed in the tumors of rectal cancer patients with or without metastatic disease and treated or not were retained by organoids derived from these different patients in a tissue-of-origin manner. [67].

#### Drug discovery and screening, immunotherapies, and personalized medicine

Drug discovery is another interesting perspective for 3D cell cultures. The development of drug candidates is a long and stringent process that goes through in vitro and in vivo tests before reaching clinical trials. During this process, hundreds of promising drugs end up being a failure [68]. Better screening of the drug candidates early in the development process may raise the success rate and represent a major economic benefit. 3D cell cultures may be a relevant screening model, especially since cells cultured in 2D and 3D do not respond similarly to treatments [69].

Firstly, the limited diffusion distance within the spheroids does not only concern biological molecules, but also therapeutic agents [70, 71]. When comparing the penetration of doxorubicin into hepatocellular carcinoma C3A cells grown in an adherent monolayer versus C3A spheroids, cell nuclei in 3D cultures integrated less doxorubicin than in 2D cultures [72]. Similar results were described using HeLa cells [73]. These results correlate with the resistance to chemotherapeutics that can be observed in vivo. Secondly, tumor hypoxia contributes significantly to the failure of conventional anti-cancer treatments. The hypoxia-inducible factor (HIF) is stabilized by hypoxic conditions that contribute to anti-cancer drug resistance, such as increasing drug efflux, inducing anti-apoptosis effects, favoring genetic instability or reducing cell proliferation [74]. Lastly, as most anti-cancer drugs target fast-dividing cancer cells (e.g. cisplatin, paclitaxel, doxorubicin, 5-fluorouracil), they are thus inefficient on the quiescent cells inside the spheroids. The sensitivity of ER-positive, HER2-amplified and triple-negative breast cancer cell lines to paclitaxel, doxorubicin and 5-fluorouracil was compared between 2 and 3D cultures [38]. The cell lines BT-549, BT-474 and T-47D that aggregated into dense spheroids showed relative resistance to paclitaxel and doxorubicin compared to 2D cell cultures. This was partially attributed to the hypoxia-induced cell-cycle arrest. Some of the cell lines that developed dense spheroids had a lower Ki-67 labelling index, which correlates with a greater number of cells in the G0 phase of the cell cycle [69].

Tumor organoids also have the potential to be an in vitro avatar for patient tumors by reproducing their molecular and phenotypic heterogeneity. In this sense, tumor organoids could be used to predict responses in individual patients in a personalized medicine manner. Ganesh et al. were able to generate 65 organoids derived from RC patients and observed a correlation between clinical and tumoroid responses to chemo- and radiotherapies. They further established an in vivo model of RC organoid xenografted mice that reproduced cancer progression. Although the cohort is small, it is very promising for evaluating patients’ responses to therapeutics and adapting their treatments [67]. This was further validated by Pasch et al*.* with tumor organoids derived from various cancers (i.e., breast, colorectal, lung, neuroendocrine, ovarian, pancreatic and prostate), biopsy sample types (i.e., core needle biopsies, paracentesis or surgery) and clinical settings (i.e., patient underwent chemotherapy and/or radiotherapy or not) [75].

Immune cells can be found in the tumor microenvironment and cancer cells often hijack their pathways to create an immunosuppressive environment that will protect them. Of all the types of chemotherapy, immunotherapy is a rapidly progressing field that also benefits from 3D cultures. Using T cells obtained from peripheral blood mononuclear cells (PBMC) and tumor cells from resection taken from the same patients, Dijkstra et al. developed a co-culture platform of patient-derived autologous T cells and mismatch repair-deficient CRC or non-small lung cancer (NSLC) organoids. With this co-culture model, they successfully expanded circulating tumor-reactive T cells that could potentially be used for adoptive T cell transfer [76]. Tumor spheroids can also help to evaluate anti-tumor drug-induced immune responses. For example, exposing spheroids from CRC cell lines co-cultured with Vδ2 T cells from healthy donors to Zoledronate or Cetuximab triggers Vδ2 T cells to kill CRC cells [77]. Following their unprecedented efficiency against hematological malignancies, the use of chimeric antigen receptor T (CAR-T) cells has recently been approved by the U.S. Food and Drug Administration (FDA) as an immunotherapy against B-cell acute lymphoblastic leukemia [78] and diffuse large B cell lymphoma [79]. CAR-T cells are genetically engineered T cells that target tumor-specific antigens. To evaluate the lethal properties of CAR-T cells that target the EGFRvIII variant commonly found in glioblastomas, Jacob et al*.* produced glioblastoma organoids (GBOs) that retain the cellular heterogeneity of the patient tumors from which they are derived. When co-cultured together for 72 h, they observed that CAR-T cells were able to infiltrate the organoids and specifically kill the EGFRvIII^+^ cancer cells, while ignoring the EGFRvII^I−^ cells. These results underline the potential of GBOs for measuring antigen-specific CAR-T cell treatment responses depending on the patients [80].

Most drug candidates that are successful in preclinical in vivo models do not lead to efficient drugs when assessed in humans [81, 82]. This underlines the fact that, even if animals are an unavoidable step in setting up clinical trials, species specificity is important. Concerning what has been discussed above, 3D models could help ensure the 3Rs (Replacement, Reduction and Refinement) by eliminating drug candidates that do not work in vitro in 3D cultures and by reducing the number of animals used.

## Types of 3D culture and their application in oncology research

When talking about in vitro 3D cultures, it is important to clearly define each model. Firstly, the 3D culture can be homotypic (only one type of cell is present) or heterotypic (two or more types of cell are present) and are called monoculture and co-culture respectively. Secondly, the cells used can be either established cell lines or primary cells. Established cell lines have acquired homogeneous genotypes and phenotypes following multiple passages. They are easier to handle, but less representative of the tissue from which they originate. On the other hand, primary cells are obtained directly from the tissue of a donor and better represent the biological variability between individuals. However, some of these primary cells may have a limited lifespan and be harder to maintain in culture. This could be the case for the cells present in the tumor microenvironment that do not replicate indefinitely, like CAFs, or stem cells which can differentiate in vitro. It should however be noted that huge improvements have been made in the culture and preservation of organoids, even making it possible to establish organoid biobanks of various cancers such as CRC [83], breast cancer [84], prostate cancer [85], lung cancer [86] or glioblastoma [80]. Finally, 3D cancer models can be categorized according to the method used to produce the 3D cultures and their final organization: (1) organ-slice cultures, obtained from cultured sections of tumor tissues [87, 88]; (2) multi-layered cell cultures, cultures of adherent cells with the property of growing in multilayers after confluence [89, 90]; (3) spherical models corresponding to tumor cells growing in spheres. Spherical models have now been used for several decades but a precise nomenclature of the different types of 3D model has not been clearly established and some terms are used interchangeably in the literature [91]. Here, we will focus on spherical models, which include spheroids and organoids, and which can be generated thanks to liquid-, scaffold-based 3D culture methods or other techniques such as microfluidic platforms or bioprinting.

### Liquid-based 3D culture systems

The main characteristic of liquid-based 3D culture systems is that cell adherence to the substrate is restricted by coating the culture vessel, using gravity, or by creating fluid movement to stop the cells from settling at the bottom of the culture vessel. In these conditions, cell–cell interaction is supported, and cells can aggregate. Therefore, liquid-based 3D culture systems are mostly used to generate spheroids, whose inherent morphology reproduces the physical and chemical gradients observed in solid tumors such as pH, oxygen gradient and soluble factors. Because of these particularities, spheroids may be good mimics of avascular tumors and micrometastases. Tumor growth can be divided into two phases: avascular and vascular. The avascular phase corresponds to tumors that receive nutrients and oxygen from the surrounding tissue, limiting their growth. These avascular tumors have cellular heterogeneity similar to that observed in spheroids cultured in vitro, with a necrotic core surrounded by dormant or slow-cycling cells and a ring of fast-dividing cells at the periphery [46]. Micrometastases are small clusters of cells (between 0.2 and 2 mm in size) that spread from the primary tumor and seed to other tissues [92]. Micrometastases are mostly avascular and require an angiogenic switch to grow into macrometastases (size > 2 mm) [93]. In the last two decades, a theory concerning the existence of a subpopulation of cancer cells known as cancer stem-like cells (CSCs) has emerged [94–96]. Even if it remains a highly debated topic in the scientific community [97], there is evidence of the existence of cancer cells with self-renewing [94], differentiation [96], and tumorigenicity properties [95], much like healthy stem cells. CSCs seems to contribute to treatment resistance and may thus be important targets for new anti-cancer therapies [94–96]. As for normal stem cell, CSCs can be identified and enriched by using a sphere-forming assay, which involves liquid-based 3D culture methods [98].

Liquid-overlay 3D culture models are for the most part simple to use, relatively low-priced, and are easily scalable for high throughput screening (HTS). Moreover, spheroids generated in a liquid environment are exposed to similar conditions (culture media density of around 1 g/cm^3^ and viscosity of 0.7–1 mPa s) as blood compartments (viscosity of 3.5–5.5 mPa s and density of 1.0–1.1 g/cm^3^) [99–102]. Otherwise, this liquid surrounding is very different from the environment that surround tumors in vivo, which should be taken into consideration when designing an experiment.

#### Liquid overlay culture

**Fig. 2:**
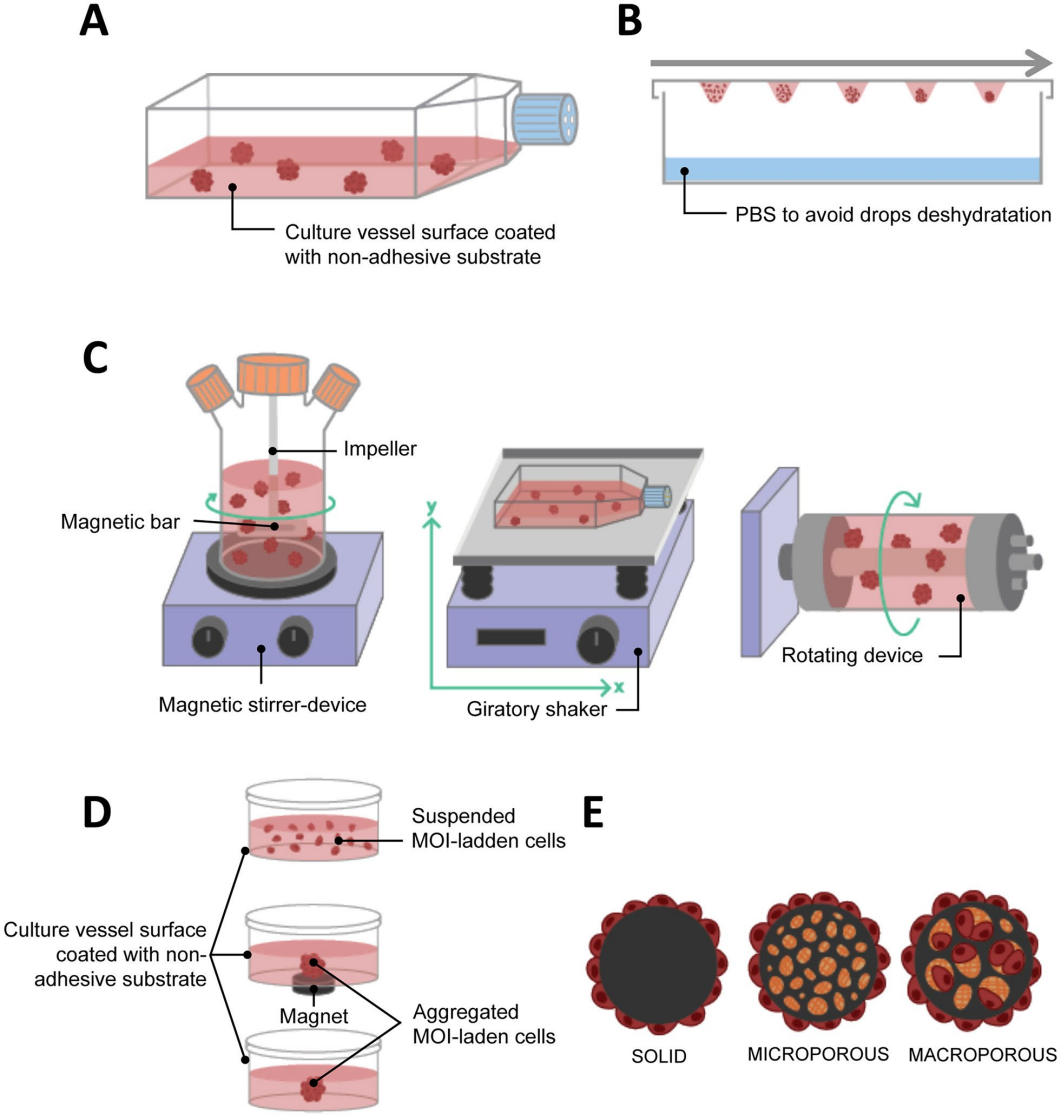
Liquid-based 3D cultures. **A** Liquid overlay; **B** Hanging drop; **C** Agitation-based: spinner flask (left), gyratory shaking (middle), rotary cell culture system/rotating wall vessel (right); **D** Magnetic levitation; **E** Microcarrier beads

Liquid overlay culture is the simplest technique for producing spheroids. Cells are seeded on a non-adhesive surface, which promotes cell–cell adhesion, leading to spontaneous spheroid formation (Fig. 2A). To create this non-adherent condition, the culture vessels can be coated with substrates such as agarose or poly-hydroxymethyl methacrylate [103–106]. Non-adherent culture vessels (with low attachment properties) are now commercially available (e.g. Greiner CELLSTAR^®^, Corning^®^ Costar^®^ Ultra-Low Attachment Surface, Nunclon™ Sphera™). The liquid overlay culture method does not require any specialized equipment and is easily implementable. It can be used to evaluate the stemness of cancer cells through the spheroid formation assay. Zhang et al*.* observed that 64% of the samples from patients with breast cancer and who underwent therapies were enriched with the ROR1 orphan receptor, which is known to be associated with cancer stemness. When cultured with the liquid overlay technique, ROR1^high^ samples had a better capacity for forming spheroids than ROR1^low^ samples. This data was supported by other functional assays such as invasion, engraftment in immune-deficient mice, and survival after treatment, confirming the stemness characteristics of the ROR1^high^ samples, which may therefore be a target of interest [107].

**Fig. 3:**
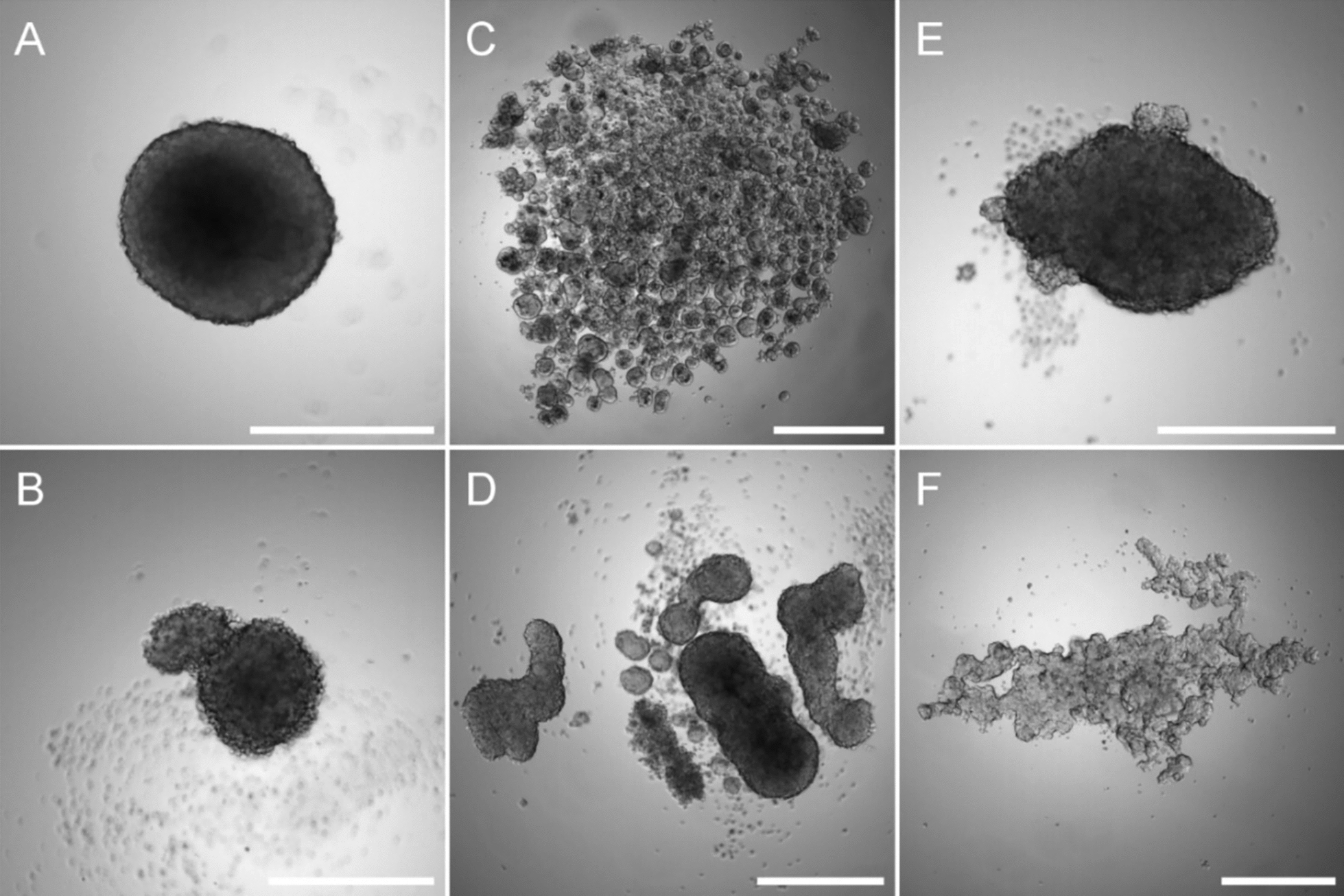
Liquid overlay technique culture. Osteosarcoma MNNG/HOS (**A**) and SAOS-2 cells (**B**), colorectal adenocarcinoma Caco-2 (**C**), colon cancer HT29 (**D**), glioblastoma U251 (**E**) or prostate carcinoma LnCaP (**F**) cells were seeded into a 96-well low-attachment plates and cultured for 7 days. Scale bar corresponds to 500 µm

However, not all cells are capable of spontaneously forming spheroids when cultured under non-adherent conditions (Fig. 3) [108]. Therefore, to promote spheroid formation some adjustments may be required, such as adding extracellular matrix components to the medium [109], coating the culture dish with bioactive materials such as hyaluronic acid, laminin or poly-d-Lysine [110–112], or using defined medium supplemented with growth factors and cytokines [113–115]. The major limitation of the liquid overlay culture method is the variability of the size and/or spherical morphology of the spheroids. In this way, extensively modulating the initial cell number, or using U-bottom or micropatterned plates such as Agrewell plates (Corning™) may be necessary to obtain reproducible spheroids.

#### Hanging drop

The hanging drop culture method relies on the use of gravity for the formation of spheroids and has been used to differentiate embryonic stem cells into embryoid bodies [116]. A cell suspension volume of less than 30 µL is pipetted onto the surface of a non-coated culture dish, usually the cover, which is then inverted. The drop will not spread on the surface thanks to gravity and surface tension allowing a spheroid to be produced at the bottom of the drop (Fig. 2B). It is possible to improve spheroid formation by using media additives such as methylcellulose and/or collagen [117]. The hanging drop culture method has multiple advantages, including its simplicity of implementation since it does not require expensive tools. It also promotes cell–cell contacts with few cells. Used as early as 1910 by R. Harrison, who cultured neuronal cells [20], it has since been widely used in cancer 3D cultures and is as a consequence a well-documented technique for different cancer cells, including breast [108, 118], prostate [119] and ovarian cancer [120]. In the case of ovarian cancer, liquid-based 3D culture methods are especially relevant since ovarian cancer cells often metastasize as multicellular aggregate in ascites that accumulate in the abdomen. Using hanging drop to generate epithelial ovarian cancer spheroids, Al Habyan et al*.* showed that cancer cells can detach spontaneously from the primary tumor as clusters that are less sensitive to apoptosis. They validated these observations in vivo [121].

At the same time, the technique also evolved and hanging drop platforms have been developed for high-throughput 3D spheroid cultures [122]. However, this method has several drawbacks. Even though the hanging drop method makes it possible to better control spheroid formation than other methods using larger volumes of cell suspension, it could prove difficult to handle the change of media or treatment of the spheroids with drugs if the spheroids are not transferred to a non-adherent culture dish after assembly.

#### Agitation-based

Another method for producing spheroids is to culture cells in a liquid environment which is in continual agitation, thus preventing their adherence to the culture recipient and increasing the collision between cells. Under these conditions, cells tend to spontaneously aggregate into spheroids. To create the agitation either the media is in continual movement through stirring or the culture bottle is in continual movement.

Stirring bioreactors include spinner flasks. Spinner flasks are three-neck flasks that can contain hundreds of milliliters of cell suspension. Gas exchanges are possible through the two side arms with filter caps. Fluid movement is produced by an impeller linked to a magnetic bar and the speed of the rotation is controlled by the magnetic-stirrer device (Fig. 2C left). The main problem with the spinner flask technique is the high shear stress associated with liquid movement, which may impede the culture of certain cell types. Because the spinner flask technique makes it possible to produce large size spheroids with a hypoxic core, various studies on oxygenation have been conducted [123, 124]. Durand and Sutherland used spinner flasks to produce spheroids of V79-171 B CHO and compared their response to radiation with single cells [125]. They observed that spheroids were more resistant to radiation damage and suggested both that cellular response can differ depending on the microenvironment conditions and that cell–cell contact could help in radiation damage repair. Co-culture is also possible with spinner flasks, and a heterotypic spheroid culture model containing FaDu head and neck squamous cell carcinoma cells and peripheral blood mononuclear cells was developed to evaluate the efficacy of immunotherapy with catumaxomab binding to CD3 and EpCAM [126]. The second method for creating liquid movement is by shaking the culture flasks. The easiest way is to use an orbital shaker that will shake the culture vessel in a 3D gyratory motion (Fig. 2C, center). The fluid movement effectively produces shear stress, but it can also help mimic in vivo conditions. Along those lines, Masiello et al*.* used this gyratory motion to reproduce the fluid shear stress that primary ovarian spheroids undergo during the transcoelomic metastasis process [127].

The rotary cell culture system (RCCS) or rotating wall vessel (RWV) was designed in the 90 s by NASA engineers to study cell cultures in a microgravity environment. This rotary bioreactor consists of a cylindrical vessel that spins slowly around a horizontal axis, thus subjecting the cells it contains to continuous free fall. In this condition, the cells are maintained in suspension and can aggregate into spheroids (Fig. 2C, right). This technique has the advantage of creating low shear stress. One of the first scientific articles published using the RCCS/RWV method described the growth pattern of several human tumors (e.g. glioma, prostate, urinary, bladder and breast cancer, and metastatic brain tumors) [128]. More recently, McNeill et al*.* showed the advantages of such 3D culture systems compared to standard monolayer cultures in malignant bone disease, and cultured viable osteosarcoma patient-derived xenografts for up to 8 days [129]. By developing a co-culture model involving osteogenically enhanced human mesenchymal stem cells and DDK1-overexpressing MOSJ cells, or Dkk-1 positive patient-derived xenografts, they were able to reproduce the mechanisms of osteo-inhibition observed in malignant bone disease. The RCCS/RWV technique, compared to other liquid-based 3D cultures such as hanging drop and magnetic levitation, makes it possible to produce a higher number of spheroids from various cell lines that are larger than 500 µm. These spheroids can then be distributed into 96-well plates for drug screening [130]. This highlights the potential for RCCS/RWV to generate spheroids for HTS. However, microgravity models should be used carefully, as such conditions do not translate well physiologically in humans.

Thanks to agitation-based techniques, large numbers of 3D spheroids can be produced extensively as fluid movement participates in the transport of nutrients and oxygen to the spheroids and aids with waste disposal. However, these rotation devices present certain limitations such as the variability in spheroid size and shape within a culture vessel [130] and the impossibility of following in real time the formation of the spheroids because of the continuous movement. To increase homogeneity in spheroid size, microcarrier beads have been used. This method cannot be directly used for HTS because of the continuous stirring, but it remains useful for mass production of spheroids. This technique requires specific equipment that can be expensive and challenging to properly assemble in the case of the RCCS/RWV method.

#### Microcarrier beads

Microcarrier beads are usually small spheres ranging from 100 to 300 nm in size that can be of different materials, such as plastic, glass, silica, cellulose or gelatin and coated with protein (collagen, fibronectin) or polysaccharide [glycosaminoglycans (GAGs), dextran]. Mainly used in large-scale cell culture production, they have been developed in oncology research to support anchorage-dependent cells that do not spontaneously aggregate to form spheroids, especially in 3D agitation-based models such as spinner flasks and RCCS/RWV. There are three types of microcarrier: solid, microporous and macroporous (Fig. 2E). With solid microcarriers, cells grow in a monolayer on their surface, and it is in fact identical to 2D adherent cell culture, except for the spherical, rather than flat, configuration. Cells growing on the surface of microporous carriers are effectively still in an adherent monolayer similar to 2D culture, but they are able to secrete ECM inside the pores of the microcarriers, creating an environment on the inside that is different to that on the outside. Finally, macroporous microcarriers with a pore diameter of more than 10 µm allow cells to invade the macropores and proliferate to form 3D cultures [131].

Microcarrier beads can be a solution if a cell type does not spontaneously form spheroids in liquid 3D culture systems, and in particular in dynamic 3D systems such as spinner flasks or RCCS/RWV [129, 132], but the procedure for harvesting the cells may be difficult, especially for cells cultured with macroporous microcarriers. Moreover, if the microcarrier used is large and made of small pores, it can be a hindrance for the diffusion of nutrients and signaling molecules to some cells. Finally, some materials used to produce microcarriers can limit microscopic observation.

#### Magnetic levitation

The 3D cell cultured using the magnetic levitation method was developed in 2010 [64] and relies on biocompatible magnetic iron oxide (MIO) nanoparticles to bio-assemble cells into spheroids. The technology is currently developed by N3D Biosciences and commercialized by Greiner Bio-One Ltd. Cells that are incubated using MIO nanoparticles assimilate them. Brief exposure to a magnetic field promotes cell aggregation and spheroid formation, coupled to cell–cell interaction and ECM synthesis (Fig. 2D). This is usually done in multi-well plates, and it produces a single spheroid per well. Pan et al*.* used magnetic levitation to highlight the involvement of miR-509-3p in attenuating spheroid formation in six ovarian cancer cell lines. The authors suggested that this microRNA may be a potential therapeutic drug that could disrupt the metastasis process in epithelial ovarian cancer that relies on the spreading of cancer spheroids into the peritoneal fluid [133]. Noel et al*.* developed a downstream metabolic assay from spheroids formed from the co-cultures of pancreatic cancer cells and CAFs [39]. This technique has the advantage of facilitating rapid and gentle cell aggregation and does not require an artificial substrate, or specialized media or equipment except for the magnets and MIO. In addition, it is not limited to a specific cell type. Co-cultures can be carried out aggregating numerous different cell types into spheroids [39, 40]. It is even possible to control to some extent the organization of the cells inside the spheroid at the beginning of the culture, by adding each cell type at different times during magnetic bio-assembly. The first cell type will undergo the magnetic action of the magnet and aggregate. Then, the second cell type is added, and cells aggregate around the previous layer and so on, creating a multi-layered spheroid [134]. The fact that each spheroid is cultured in a single well and undergoes the same magnetic field as the neighboring spheroids makes possible highly reproducible experiments. Finally, this method is suitable for HTS. However, magnetic levitation has some disadvantages. Firstly, it has been proven that the MIO does not have a direct effect on cell behavior [64, 135], however magnetic fields of more than 30 mT affect angiogenesis [136], tumor spheroid growth [137], and cell migration [138]. Moreover, the magnetic nanoparticles color the cells brown because of the iron oxide, which can hinder assays involving colorimetric reagents that generate a brown product upon reaction with an enzyme, such as 3,3′-Diaminobenzidine or *o*-phenylenediamine.

### Scaffold-based 3D culture systems

Scaffold-based 3D culture systems act as a structural support for cell attachment and growth and thus reproduce the ECM to a certain extent. The porosity, solubility, compliance and composition of scaffolds affect the cellular response [139] and consequently, the choice of biomaterial and synthesis method used to produce the scaffold will depend on the origin and stage of the cancer, the microenvironment cells, and the investigations carried out. A change in ECM composition has been shown in different stages of colorectal cancer, with an increase in the expression of type I collagen, MMP-2 and MMP-9, and a decrease in the expression of type IV collagen and TIMP-3 in the late stages. The ECM of colorectal cancer is associated with a higher proliferation rate for cancer cells compared to the ECM of normal colons [140]. Similarly, different extracellular matrix signatures were detected between normal colons, primary colon tumors and their metastases in the liver [141]. To address the multiple types of ECM observed in vivo, a variety of scaffolds have been developed. The scaffolds used for 3D cell cultures are either of natural or synthetic origin (Table 1) and the methods used produce a hydrogel, porous or fibrous scaffold (Table 2). Hydrogels are polymer networks containing a high percentage of water that can be obtained from natural sources or be synthesized. Hydrogels are synthesized when hydrophilic polymers undergo gelatinization following physical and/or chemical crosslinking. By reproducing the hydrophilic and gel-like structure of the natural ECM, hydrogels recreate an in vivo-like environment to support cell growth in vitro. Porous scaffolds contain pores around 100 µm in diameter. Fibrous scaffolds are composed of polymer fibers (Table 2).

**Table 1:** List of natural and synthetic polymers used for the production of scaffolds

	Type of polymer	Subtype of polymer	Advantages (+)/disadvantages (−)
Natural scaffold	Protein-based	Collagen Elastin Fibronectin Fibrin Gelatin Silk fibroin	** + **Biocompatibility ** + **Inherent bioactivity **−** Complex structure **−** Difficulties to control the stiffness, the degradability and the bioactivity **−** Inter-batch variability **−** Technical approach relatively expensive
	Polysaccharide-based	Glycosaminoglycan (hyaluronic acid, chondroitin sulfate) Alginate Chitosan	
	Decellularized ECM		
Synthetic scaffolds	PEG pHEMA		** + **Well-defined structure ** + **Highly reproducible ** + **Possibility to modulate the biochemical and chemical properties **−** No inherent bioactivity **−** Require functionalization for cell adhesion − Lower biocompatibility than natural scaffolds
	PVA SAPs	RADA16-I (commercially available as Puramatrix^®^) Fmoc (commercially available as Biogelx^®^) H9e FEFK MAX1	
	Aliphatic polyester	PCL PGA PLA PLGA	

*ECM* extracellular matrix, *PEG *poly(ethylene) glycol, *pHEMA* poly(2-hydroxyethyl methacrylate), *PVA* poly(vinyl alcohol), *SAPs* self-assembling peptides, *PCL* polycaprolactone, *PGA* poly(glycolic acid), *PLA* poly(lactic acid), *PLGA* poly(lactic-*co*-glycolic acid

**Table 2:** Methods of production of scaffolds and their advantages and disadvantages

Method of production	Description of the mechanism	Advantages (+)/Disadvantages (−)	References
Lyophilisation/freeze-drying	Polymers are solubilized in solvent, before being subjected to gelation sublimation of solid polymers (gel or foam) followed by freeze drying under vacuum	+ High porosity and pore interconnectivity – Small pore size − Irregular porosity − Time consuming process (days) − Residual solvent that may be harmful to cells − High energy-consuming	[142, 143]
SCPL	Insoluble salt particles are added to a solution of polymers solubilized in solvent. After solvent evaporation, a composite of polymers embedded with salt particles is obtained. Repeated washing of the composite with water allows the salt elimination and then the formation of a porous scaffold	+ Simple + Reproducible + No specific instrument required − Limited interconnectivity − Time consuming process (days) − Residual solvents that may induce cell damages	[144]
Gas foaming	Can be done chemically by: i) producing hydrophobic gas bubbles in liquid solution of polymers; ii) physically by subjecting a solid polymer to a high pressure gas that can dissolve into it and expands when the pressure is reduced, thus producing cavities when the bubbles collapse. It can be associated with SCPL	+ High porosity + Controlled pore size + Solvent-free − Limited interconnectivity	[145–147]
TIPS	Relies on the change in thermal energy to transform a homogeneous mixture of polymer and solvent into a multiple-phase system, composed of a polymer-rich phase (solvent-poor phase) and a polymer-poor phase (solvent rich phase). The solution is quenched below the freezing point of the solvent, and the solvent is removed by freeze-drying	+ Easily implementable + High interconnectivity + Easy modulation of pore size and structure − Time consuming process (days) − Residual solvents may induce cell damages − High energy-consuming	[148, 149]
Electrospinning	A charged liquid with a voltage high enough to counteract surface tension will stretch and erupt into a jet. It will solidify into a fibre when projected on a collector	+ High porosity + High interconnectivity + Low cost + Most soluble polymers can be used + Mimic the fibrillar structure of ECM − Complex generation of 3D structure − Residual solvents that may induce cell damages − Small pores that lead to poor cell infiltration and distribution − Low mechanical strength	[150, 151]
Self-assembly	Spontaneous assembling of monomers into supramolecular nanostructures after exposure to pH or temperature modifications or enzymatic treatment	+ Different types of structure can be generated depending on the synthesis conditions + Easy to functionalize with various molecules + Less toxic because does not require cross-linker reagents + Low cost and rapid syntehesis − Difficult to control size of the self-assembled nanostructure − May be unstable under liquid conditions	[152, 153]
Rapid prototyping	Describes a group of manufacturing processes (e.g. stereolitography, 3D printing, selective laser sintering) that enables fabrication of scaffold layer by layer with precise spatial organization from a computer aided design (CAD)	+ High control on pore size, porosity, and interconnectivity + Good resolution + Good reproducibility − Expensive − Time-consuming (creation of the design) − Potential wasting of polymers − Potential cytotoxicity of the polymers used	[154]

*SCPL* solvent-casting and particulate-leaching, *TIPS* thermally induced phase separation

#### Scaffolds of natural origin

Natural scaffolds include protein-based, polysaccharide-based and decellularized ECM scaffolds. Collagen and its derivatives gelatin and gelatin methacryloyl (GelMA) scaffolds belong to the protein-based systems. Collagen is the main fibrous protein in the ECM in connective tissue, representing one-third of the whole-body protein content [142]. Collagen scaffolds have been used for numerous 3D cultures of cancer cells such as breast [143] and ovarian cancer [144], pancreatic ductal adenocarcinoma [145], head and neck squamous cell carcinoma [146] and liver cancer [147].

Matrigel™ is a complex mixture of basement membrane proteins, growth factors and cytokines that are secreted by Engelberth-Holm-Swarm mouse sarcoma cells. Its main proteins are laminin, collagen IV, heparan sulphate and entactin [148]. Due to its cancer origin, Matrigel™ has been extensively used in oncology research, particularly for studying cell invasion and metastasis, CSCs and cancer resistance [149]. Recently, Zhang et al*.* developed a dumbbell model to directly observe the physical interaction between CAFs and cancer cells. Fibroblasts and cancer cells were suspended in Matrigel™, seeded in two separated droplets, and linked to each other by a Matrigel™ causeway. This model was validated using BHK-21 fibroblasts co-cultured with either CaKi-1 kidney carcinoma cells, HeLa cervical cancer cells, A375 human melanoma cells or A549 lung adenocarcinoma cells [150]. Matrigel™ has been also used to identify CSCs and study their characteristics and properties [151]. Bodgi et al*.* used this method to assess the radiosensitivity of bladder cancer in vitro. They observed that cancer cells did not respond in the same way to irradiation when cultured in monolayers compared to 3D cultures in which cells were predominantly more resistant to irradiation. They hypothesized that 2D cultures did not favor CSC generation in contrast to 3D cultures in Matrigel™ that may be responsible for the reduced radiosensitivity [152]. Moreover, when exposed to doxorubicin, MDA-MB-231 spheroids cultured in Matrigel™ were less sensitive to drugs than cells cultured in PuraMatrix™ (a synthetic peptide hydrogel that is devoid of animal-derived materials), which underlines the role of ECM proteins in chemoresistance [48]. Finally, Matrigel™ is the most commonly used matrix to support organoid growth [153–155]. However, using Matrigel™ can result in high and unequal background signals between batches because of contaminants and batch-to-batch variability. Depending on the downstream analyses, different strategies have been developed to minimize these background signals. For example, acetone precipitation of peptide digest from Matrigel™-embedded samples before liquid chromatography with tandem mass spectrometry (LC–MS/MS) makes it possible to increase the number of spectra identified as peptides. This method was applicable to large- and small-sized samples obtained from CRC patient-derived organoids. It thus improved the potency of phosphoproteomic studies as assays for the profiling of individual phosphorylation patterns that may be the mirror of cancer progression and treatment response in patients [156]. Matrigel™ is also an issue for matrix-assisted laser desorption/ionization mass spectrometry imaging (MALDI-MSI). MALDI-MSI is a technology that uses a molecule’s mass-to-charge ratio for its identification without any other probes, making it possible to image a thousand molecules (i.e. peptides, lipids, proteins, glycans, and metabolites) in a single experiment. Overlapping signals between Matrigel™ and organoid cells can thus be detected. By adding a centrifugation step at 4 °C, Johnson et al*.* successfully extracted the organoids from the Matrigel™, and could then transfer them to a gelatin mold that did not generate background noise with MALDI-MSI. With this method, it was even possible to do subject the organoids to HTS. The organoids extracted from the Matrigel™ were transferred to a microarray grid composed of micro-wells. After centrifugation, all organoids were thus aligned on the same z-axis, which makes it possible to have them all in a single section instead of having to section them one-by-one [157].

A second type of natural polymer is composed of polysaccharide-based scaffolds. GAGs are linear polysaccharides that are capable of attaching covalently to ECM proteins or with other GAGs, and play major roles in stabilizing the ECM. Their structures (chain length and sulfation patterns) differ between cancerous and healthy tissues, and between primary and metastatic tissues [158]. Hyaluronic acid (HA)-based hydrogels are the most used in oncology research. They have been used to elaborate 3D models [159–161], to study cell behavior in response to microenvironment modifications [162–164] or for high throughput screening [165, 166]. Alginate and chitosan are also polysaccharides respectively found in the cell walls of brown algae and the exoskeletons of arthropods. Liu et al*.* created a composite collagen-alginate hydrogel whose stiffness was comparable to the matrix found in human breast tumors and studied the functional impacts of their structural (e.g. gel porosity) and biological modifications (e.g. addition of a chemotactic gradient) on tumor invasion [167]. By using a collagen-HA composite scaffold, Rao et al*.* analyzed the behavior of glioblastoma cells. They observed that the nature of the collagen used in the composite scaffold influenced cell morphology, and that the HA concentrations led to the regulation of cancer cell migration, highlighting the major signaling role of the cancer microenvironment [139]. Maloney et al. also used a collagen-HA composite scaffold as a bio-ink to bioprint organoids in a HTS platform for drug screening. This bio-ink was more efficient in generating spheroids than the commercially available HyStem HA-polyethylene glycol (PEG) hydrogel (https://www.ncbi.nlm.nih.gov/pmc/articles/PMC7074680/), which is a composite of natural and synthetic hydrogels that has been used to expand stem cells [168].

It remains challenging to reproduce the in vivo microenvironment by crosslinking several polymers into a single scaffold. Tissue decellularization is a good alternative for producing an ECM similar to the original tissue/organ. During this process, all cells are removed from the tissue, and only the ECM and its structure remain. Decellularization is done by physical (e.g. temperature, electroporation, hydrostatic pressure), chemical (e.g. ionic and non-ionic surfactants, acids and bases) and enzymatic (e.g. trypsin, nuclease, dispase) means [169]. By decellularizing normal and tumor tissues resected from colorectal cancer patients, Pinto et al*.* showed that the tumor microenvironment induced M2 macrophage polarization, which are known to be anti-inflammatory and pro-angiogenic agents that promote cancer progression [170]. Hoshiba and Tanaka showed that using decellularized ECM derived from different malignant stages affected the 5-FU sensitivity of HT-29 colorectal cancer cells. When cultured in late-stage cancer decellularized ECM, HT-29 were more resistant to 5-FU treatment compared to low malignant derived decellularized ECM [171].

Since all these scaffolds come from natural sources, inter-batch variability is an issue and some of the polymers can be hard to extract in large quantities (e.g. collagen). Moreover, their structure is complex, not well defined and could lead to non-reproducible properties from one scaffold to another. Therefore, scientists developed synthetic scaffolds to reduce the cost of production and to limit the variability in the hydrogels produced.

#### Synthetic scaffolds

In contrast to biopolymers of natural origin, the chemical composition of synthetic scaffolds is fully controlled. It is possible to fine-tune their biochemical and mechanical properties (i.e. stiffness, biodegradability and bioactivity) and these scaffolds are highly reproducible.

PEG is a synthetic polymer that swells to form a hydrogel in a few minutes when exposed to an aqueous solution. Incorporating biochemical and biological functionalities into the PEG polymer backbone makes it possible to directly study their impact on cancer cells [172]. Sieh et al*.* used a PEG-based hydrogel, functionalized with the arginine-glycine-aspartic acid (RGD) motif (which provides binding sites for cells through integrin) and matrix metalloproteinase (MMP) cleavage sites (which allows cells to degrade the gel). With this model, the authors showed the effect of 3D cultures on the morphology, gene expression and protein synthesis of LnCaP prostate cancer cells compared to adherent monolayers [173]. Another recent study showed that by changing the chemical and mechanical properties of a PEG-based hydrogel, it was possible to control the phenotype state of MDA-MB-231 breast cancer cells, directing them toward a highly proliferating, moderately proliferating, or dormancy phenotype [174]. PEG hydrogel was also hybridized with natural polymers such as collagen [175–177], chitosan [178–180] or Matrigel™ [181] to improve the biocompatibility of the scaffold.

Other commonly used synthetic materials for scaffold production are aliphatic polyesters such as polycaprolactone (PCL), poly(glycolic acid) (PGA), poly(lactic acid) (PLA) or poly(lactic-co-glycolic acid) (PLGA). As with PEG, polyesters have high biocompatibility and tunability [182]. The major difference between these polyesters is their biodegradation rate. There is an inverted relationship between hydrophobicity and degradability. The more hydrophobic a polymer is, the less degradable it will be. A list of polymers with slow to fast degradability can then be defined: PCL < PLA < PLGA < PGA [183]. When cultivated into a PCL scaffold, TC-17 Ewing sarcoma (EWS) cells exhibited proliferative rates and anti-cancer drug responses similar to in vivo EWS tumor xenografts, in contrast to cells cultured in 2D [184]. PCL scaffolds also allowed CAFs to retain their pro-inflammation properties, while these properties were lost in 2D cultures. When xenotransplanted in vivo, these 3D cultured CAFs promoted inflammatory cell infiltration at the tumor site and the invasiveness of cancer cells [185]. In another study involving PCL scaffolds, organoids were generated by transient culture of CAFs on the scaffold before removing it, followed by culture of primary breast cancer cells [186]. CAFs deposited ECM on the PCL scaffold, encouraging cancer cell adhesion, survival and proliferation. On the other hand, cancer cells grown on the PCL scaffold without pre-culture with CAFs failed to form organoids even after 10 days in culture. In addition, this hybrid PCL-CAFs ECM scaffold made it possible to study patient-specific responses to treatment by exposing the tumoroids to doxorubicin or mitoxanthrone. This 3D culture model could therefore serve as a personalized medicine platform [186]. Girard et al*.* developed a PLGA/PGA/PEG co-polymer scaffold in which they induced the formation of spheroids from various cancer cell lines (melanoma, breast, prostate, ovarian, and lung cancers). These spheroids underwent epithelial-to-mesenchymal transition (EMT), losing the expression of E-Cadherin and acquiring vimentin expression. They also displayed higher resistance to cytotoxic drugs than cells cultured in 2D [187].

Self-assembling peptides (SAPs) are short molecules that spontaneously assemble into supramolecular nanofiber structures [188] when exposed to modified pH, temperature or enzymatic treatment [189]. SAPs have been used as carriers for drug delivery systems [190, 191], but they also show great potential for 3D cell cultures in cancer research [192]. They are highly biocompatible [193], can be easily tuned, and offer a fibrous network organized similarly to the ECM [194]. Moreover, the short length of the peptides (< 20 amino acids) renders their synthesis easy, rapid and non-expensive. Of the variety of existing SAPs (Table 2), RADA16 is the most commonly used and is commercially available under the name PuraMatrix^®^. RADA16 hydrogels make it possible to culture different types of cancer cell such as pancreatic ductal adenocarcinoma [195], breast carcinoma [150], hepatocellular carcinoma [160] and leukemia [193].

### Emerging methods for 3D tumor models

#### Microfluidic platforms

**Fig. 4:**
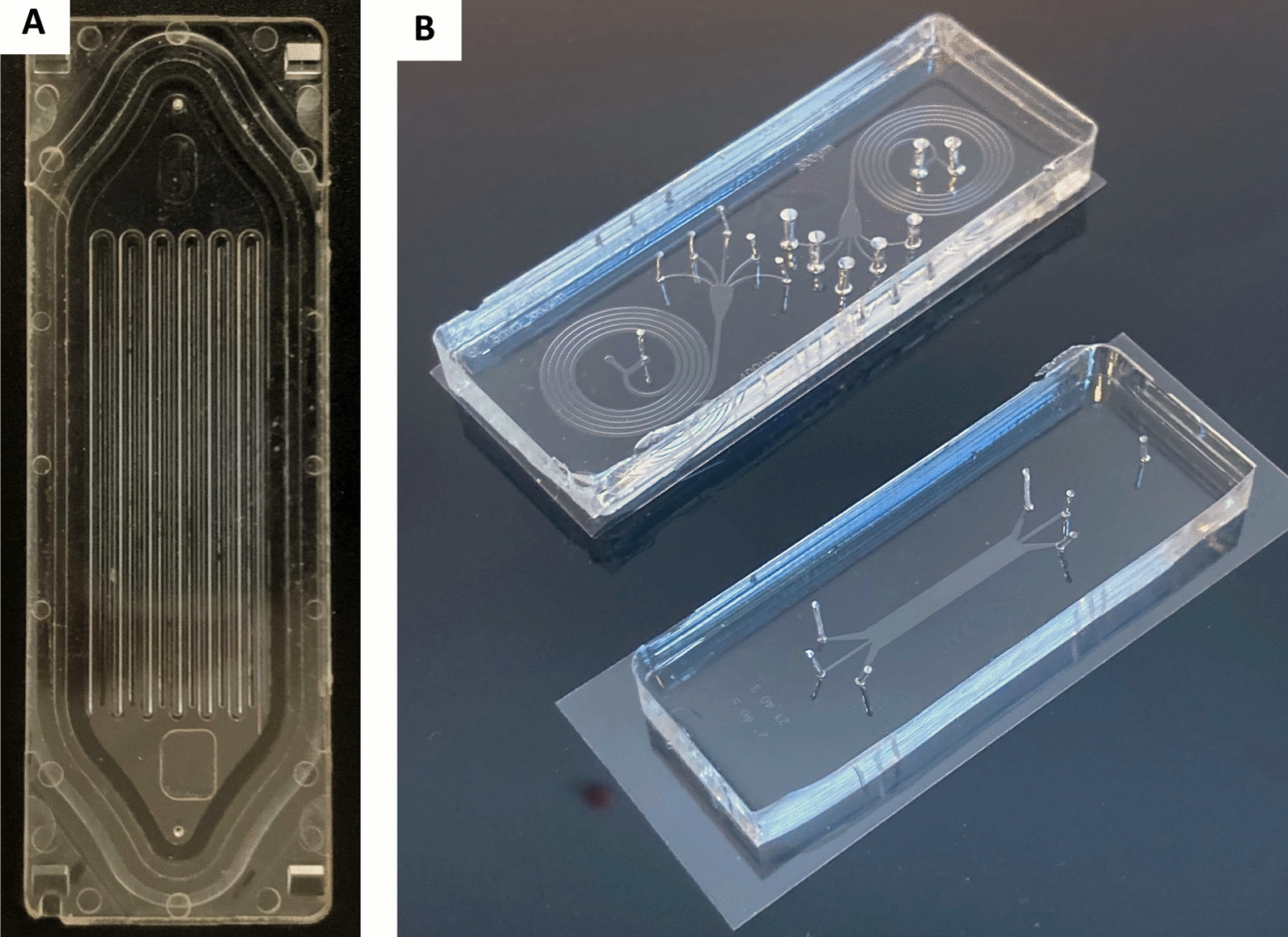
Microfluidic platforms. **A** Parsortix™ microfluidic platform for isolating circulating tumor cells based on their size and their deformability properties; **B** Image of PDMS microsystems dedicated to particle separation: spiral microfluidic systems (top); deterministic lateral displacement particle separation system (down). Both are placed on 60 × 22 mm coverslips

Microfluidic platforms are based on the manipulation of fluids, in small volumes and spaces (in the micro range) within a network of channels. This small dimension creates reproducible and predictable laminar flow that facilitates the formation of homogeneous spheroids [196]. Although microfluidic devices have been used to separate cancer cells, such as circulating tumor cells (CTCs) from blood [197], their use has been extended to cancer cell cultures either in 2D or in 3D in the past two decades (Fig. 4). 3D microfluidic platforms can take various forms, be made of different materials (Table 3), and incorporate scaffolds of multiple types to better mimic the in vivo tumor microenvironment. This variety of platforms was developed to study the wide variety of cancer types and their multiple mechanisms. Jeon et al*.* developed a co-culture 3D microfluidic model made with polydimethylsiloxane (PDMS) to study the metastatic process of breast cancer cells [198]. They investigated the ability of a bone-seeking clone of the MDA-MB-231 breast cancer metastatic cell line to extravasate into a bone-mimicking microenvironment, into a muscle-mimicking microenvironment or into an acellular collagen matrix. They observed a significantly higher extravasation rate for the breast cancer cells in the bone-mimicking microenvironment compared to the other two microenvironments, highlighting the seed-and-soil theory. With the aim of studying metastatic processes, Toh et al*.* also proposed a monoculture 3D microfluidic PDMS model [199]. These authors induced the aggregation of MX-1 breast cancer metastatic cells inside the microfluidic platform before adding a chemoattractant to stimulate cell motility. Cancer cells exhibit amoeboid-like motility (the cells are amorphous and change direction rapidly) and collective motility (cells retain their cell–cell contacts and invade as a group) that have only been observed in 3D in vitro models or in in vivo models. By using this system, Toh et al. observed in real-time the migration and invasion of the cancer cells across a collagen barrier. This approach could be highly useful in anti-metastasis drug assays. Other cancer mechanisms were studied, such as tumor angiogenesis. Recently, Miller et al*.* used a co-culture 3D microfluidic PDMS model to study endothelial sprouting induced by primary human clear cell renal cell carcinoma (ccRCC) [200]. Their model reproduced the pro-angiogenic activity of ccRCC cultured in 3D in contrast to 2D transwell assays. Moreover, pharmacological angiogenesis blockade could be then modelled, demonstrating the major value of this type of platform for screening anti-angiogenic drugs.

**Table 3:** Materials used to engineer microfluidic platforms

Material	Properties/characteristics	Advantages (+) /disadvantages (−)	References
Glass	Transparent Stiff No gas permeability Hydrophilic surface	**+ **High optical properties (no intrinsic fluorescence) + Highly reproducible + No absorption of molecules − Not permeable to O_2_ − Prone to breaking − Relatively expensive	[210]
PDMS	Transparent Soft, flexible Gas permeability Highly hydrophobic surface	+ Rapid prototyping + Low cost + Permeable to O_2_ + Low auto-fluorescence − High gas permeability can lead to evaporation − Possible absorption of small hydrophobic molecules and proteins − Poor resistance to solvents and acids/bases − Deformable **−** Require treatment to increase hydrophobic properties	[211]
PMMA	Transparent Stiff Low gas permeability Hydrophobic surface	+ Low auto-fluorescence + Lower cost than PDMS + lower evaporation rate than PDMS + More resistant to small hydrophobic molecules and proteins absorption than PDMS + Good solvent and acid/base resistance − Long culture are impossible because of the low O_2_ permeability − Require treatment to increase hydrophobic properties	[211, 212]
PC	Transparent Stiff Low gas permeability Hydrophobic surface	+ Lower cost than PDMS + Good solvent and acid/base resistance + More resistant to small hydrophobic molecules and proteins absorption than PDMS − High auto-fluorescence − The low permeability to O_2_ do not allow long term cultures − Require treatment to increase hydrophobic properties	[211, 212]
PS	Transparent Stiff Low gas permeability Hydrophobic surface	+ Lower evaporation rate than PDMS + Good acid/base resistance − Poor resistance to solvents − The low permeability to O_2_ do not allow long term cultures − High fluorescence − Require treatment to increase hydrophobic properties	[212, 213]
PU	Transparent Soft to stiff Hydrophobic surface	+ Tunable rigidity (duromoter shore hardness from A to D) + More resistant to small hydrophobic molecules and proteins absorption than PDMS + Good resistance to abrasion − Require treatment to increase hydrophobic properties	[214]

*PDMS* polydimethylsiloxane, *PMMA* poly(methyl methacrylate), *PC* polycarbonate, *PS* polystyrene, *PU* polyurethane

Microfluidics platforms have also been used to expand organoids. Pinho et al. observed that growing CRC cells in the microfluidic platform that they developed promoted organoid growth compared to traditional organoid cultures in 12-well plates. These results may be imputed to the continuous perfusion of the organoids with fresh culture media in the microfluidic platform. Moreover, this microfluidic platform was compatible with drug screening, although not in a high throughput way since the microfluidic chip has only four seeding wells [201]. However, microfluidic devices do not preclude HTS. Indeed, Schuster et al*.* developed an automated microfluidic platform for dynamic and combinatorial drug screening. Their platform could hold up to 200 samples sub-divided into 20 units of 10 wells each. Each unit could be perfused with independent fluidic conditions (i.e. drug solutions). Moreover, their device was compatible with live-cell time-lapse fluorescence microscopy for the whole duration of the experiment. With this microfluidic platform, they grew pancreatic ductal adenocarcinoma organoids from three different patients that they subjected to constant-dose monotherapy, one-shot combinatorial therapies, or sequential drug administration. They observed that the latter was the most effective therapy. In addition, it is possible to harvest the organoids when an experiment is completed for downstream analysis. Their microfluidic platform could serve as a screening platform for personalized medicine [202].

PDMS is the main substrate used for the biofabrication of 3D microfluidic platforms. PDMS is biocompatible, can be easily modeled, and is transparent, which facilitates imaging. In addition, PDMS is permeable to gases, facilitating simple gas exchanges. However, PDMS also has multiple disadvantages. It is permeable to water evaporation, which can lead to samples drying out. The porosity of the material is also responsible for high adsorption of cytokines and other signaling molecules, which could produce misleading results [203]. To overcome this disadvantage, microfluidic platforms based on polystyrene and other materials have been proposed [204]. 3D microfluidic platforms go a step further in mimicking in vivo tumors than the 3D culture models presented previously, but they require interdisciplinary collaborations (physics, biochemistry, engineering, and biology) and high-cost material/equipment.

#### Bioprinting

3D-bioprinting is an innovative approach based on automatic additive manufacturing that offers the potential of assembling tissue-like structures by controlling and positioning cells, tissues and biodegradable biomaterials within a prescribed organization to accomplish one or more biological functions [205, 206]. As such, 3D bioprinting offers flexibility in dispensing cells and biomaterials spanning the disparity between artificially engineered tissue and native tissue [35]. This flexibility facilitates the formation of complex architectures and features that may affect tissue function, which could be the stepping stone to personalized medicine [207]. For example, the construction of a “mini-brain” which includes the incorporation of different cell types capable of interacting with each other was used to test a range of chemotherapeutic agents [208]. The hydrogel-based biomaterials used in bioprinting are called bio-inks that must possess adequate viscoelastic properties to guarantee detailed layer by layer deposits, resulting in high fidelity 3D printed constructs [209]. Depending on the biomaterial present in the bio-ink, the 3D structures formed can be solidified through three different mechanisms: physical (temperature or light) [210], enzymatic [211], or chemical crosslinking (pH and ionic compound) [212]. 3D bioprinters differ through their bioprinting modalities and can thus be classified into 4 categories: (i) droplet-based bioprinting, (ii) laser-based bioprinting, (iii) extrusion-based bioprinting and (iv) stereolithography bioprinting. Each of these bioprinting modalities uses various strategies (Table 4).

**Table 4:** Bioprinting: categories, mechanism involved, advantages and disadvantages

Type	Subtype	Advantages (+)/disadvantages (−)
Droplet-based bioprinting	Inkjet-based bioprinting: either relies on Plateau-Rayleigh instability phenomenon (CIJ), or on the generation of droplets by a thermal, piezoelectric or electrostatic stimulus that overcome the surface tension force of the bioink at the nozzle (DOD) EHDJ: use back pressure to push the bioink to the nozzle tip until forming a spherical meniscus. Then, a high voltage is applied between the tip of the nozzle and the bioink, which creates an electric field that overcomes surface tension Acoustic bioprinting: the bioink is ejected from an open pool instead of a nozzle, thanks to the action of an acoustic field whose waves focalize at the pool exit and overcome the surface tension force of the bioink at the nozzle Microvalve bioprinting: a voltage applied will open the microvalve that gate the nozzle tip, and with association with a pneumatic back pressure, the bioink is ejected	+ High printing speed + Low cost + High cell viability − Require specific equipment − Low cell density printable − Low bioink viscosity − Clogging issues − Weak mechanical integrity of the construct
Extrusion-based bioprinting	Pneumatic: use of air pressure to extrude the bioink Mechanical: use of a piston or a screw to extrude the bioink Solenoid: use the effect of electric current on magnetism. A ring magnet localized around the nozzle attracts a second magnet that floats in the bioink inside the syringe barrel, thus closing the nozzle hole and preventing bioink to flow through. When an electrical pulses are generated into a coil surrounding the syringe barrel, it cancels the magnetic attraction between the ring and floating magnet, allowing the bioink to flow through the nozzle onto the substrate	+ Simplicity of the system + High scalability + Good structural integrity + High cell density printable + High bioink viscosity − Lower resolution than inkjet- and laser-assisted bioprinting (100 µm) − High sheer stress can impact cell viability − Clogging issues − Slow printing speed − Require sheer thinning bioink
Laser-assisted bioprinting	Cells in bioink: consists in a donor slide that contains a transparent layer, most often a laser energy-absorbing layer, and a layer of cell trapped in bioink. A laser goes through the transparent layer, its energy is absorbed by a metal or biopolymer layer, which creates local evaporation and the high gas pressure propels a droplet from the bioink layer onto the substrate (LIFT, AFA-LIFT, BioLP, MAPL-DW) Cells in liquid media: cells are in suspension in liquid media placed above a substrate, and a weak powered laser go through cell suspension and push the cells down onto the substrate (LG DW)	+ High cell viability + High resolution (5 µm) + Good printing speed + No clogging issues + Higher cell density printable than with droplet-based bioprinting − Low bioink viscosity − Laser exposure can lead to phototoxic damages − Metallic nanoparticles in the absorbing layer can be cytotoxic − High cost − Complexity of the donor slide production
Stereolitography bioprinting	Direct laser writing: a laser trace lines across the photopolymer surface to cure it Mask projection: use either a patterned physical or digital mask to filter light and cure a whole layer of photopolymer at once	+ Highest resolution among all bioprinting methods + Low cost + High cell density printable + No clogging issues + Good printing speed with masks + High bioink viscosity − UV and IR phototoxicity can lead to low cell viability − Few bioink compatible with stereolithography bioprinting

*CIJ* continuous inkjet, *DOD* drop-on-demand, *EHDJ* electrohydrodynamic jetting bioprinting, *LIFT* laser-induced forward transfer, *AFA-LIFT* absorbing film-assisted laser-induced forward transfer, *BioLP* biological laser processing, *MAPL-DW* matrix-assisted pulsed laser evaporation direct writing, *LG DW* laser-guided direct writing

Inkjet bioprinting is adapted from conventional inkjet printing, which delivers droplets on to a print controlled by thermal, piezoelectric, or microvalve methods [206, 213]. The droplets of solution can be positioned in a highly precise mode at high speed, allowing for the construction of complex 3D structures. Inkjet bioprinting offers some distinct advantages, such as high-speed printing of up to 10,000 droplets per second, moderately high resolution suitable for biological constructs (50–300 μm), low cost and control of the concentration of cells and growth factors in the bio-ink. However, inkjet bioprinting is restricted to low viscosity bio-inks (< 10 mPa/s), preventing the assembly of thicker vertical structures [214] since the more viscous the bio-ink, the greater the force required to eject droplets from the printing nozzle, thereby limiting its applicability [215]. Furthermore, encapsulated cells in the bio-ink increase the viscosity of the solution, thereby limiting the number of encapsulated cells tolerated in the bio-ink [216]. Due to this limitation, fabrication of thick complex tissues poses a huge challenge. On the other hand, it is a powerful printing method for generating organoids for drug screening in a high throughput and rapid manner. Jiang et al*.* developed a homemade droplet printer that involved microfluidics and 3D scaffold-based cultures. By printing Matrigel™ droplets laden with around 1,500 cells from mouse or human lung, kidney and stomach tumors loaded into 96-well plates, they produced organoids within one week with a success rate of 95%. These organoids were representative of inter-organoid homogeneity and inter-patient heterogeneity, as shown by the similar gene mutation signature and response to drugs between the organoids and the tumor they were derived from. While this organoid platform still needs improvement, especially since the number of organoids that can be produced is limited by the tumor samples, the one-week duration required to generate these organoids is far shorter than the time usually required by conventional protocols [154] and could therefore fit within a personalized medicine context [155].

Microextrusion bioprinters are extrusion-based, with bio-inks driven through single or multiple nozzles by a pneumatic (air-pressure or mechanical screw/piston-driven) dispensing system [217]. This approach is a combination of a fluid-dispensing system and an automated robotic cartesian system for extrusion and bioprinting, where bio-inks are spatially disposed under computer-controlled motion, resulting in the precise depositing of cells encapsulated within the bio-ink as micrometric cylindrical filaments making possible the desired 3D custom-shaped structures. This rapid fabrication technique provides better structural integrity compared to inkjet bioprinted constructs due to the continuously deposited filaments. Filaments are expelled mechanically using a pneumatic pump, piston, or screw to drive the fluid flow and build up, layer-upon-layer, a 3D structure using a robotic stage and a printhead capable of x–y–z directional mobility [218]. Microextrusion bioprinting typically uses soft biomaterials in the form of a hydrogel. Similar to inkjet bioprinting, the materials can be crosslinked ionically, enzymatically, chemically, or with ultraviolet light, as well as thermally [219]. The resolution of the printed filaments depends on a number of factors, including the size of the nozzle used, the flow rate of the extruded material, and the speed of the printhead while dispensing the biomaterial. The main advantage of microextrusion bioprinting is the versatility of the technique. The use of mechanical force to dispense the materials and an adjustable nozzle or needle inner diameter makes possible a high working range of material viscosities (30 mPa/s to > 600 kPa/s) and the ability to print a high concentration of cells or cellular aggregates similar to the numbers of cells seen in natural tissues [219, 220]. Higher resolution would require a smaller nozzle diameter and imposes higher shear stress on the cells, requiring higher pressure to extrude the material and this could have an effect on cell viability. Other challenges with this technique include nozzle clogging and insufficient interlayer bonding, depending on the crosslinking method. Despite these minor challenges, microextrusion bioprinting makes it possible to manufacture constructs of clinically relevant sizes and is often regarded as the most promising bioprinting technique [221, 222]. However, the homogeneity of the organoids generated when scaling up is a source of issues, even when using extrusion bioprinting. Substrates with small-sized wells make the process of printing bio-ink in the form of a bead difficult, because it spreads to the wall of the well. Maloney et al*.* suggested a new method based on extrusion bioprinting in an immersion bath made of gelatin. With the right concentration of gelatin, printing in this immersion bath allowed the bio-ink loaded with cells to remain spherical in shape until crosslinking. They were able to produce viable organoids from different cell lines and from glioblastoma and fibrosarcoma patient samples. Moreover, they proved that their method was compatible with drug screening by subjecting patient-derived organoids to three concentrations of two different drugs [223].

Laser-assisted bioprinting relies on the use of a donor slide of biomaterial covered with a laser energy absorbing layer which locally evaporates and projects the donor slide material on to the substrate [224]. This nozzle-free (and clog free) system has a significant advantage given that it is capable of depositing biomaterials containing high cell densities while maintaining high cell viability and resolution [220]. The resolution is of the order of single cells in a droplet of 20–80 μm in diameter [219]. However, laser-assisted bioprinting requires a material that is moderately low in viscosity (1–300 mPa/s) and has a fast gelatinization mechanism to achieve high fidelity in the shape of the 3D bioprinted constructs [214]. Furthermore, preparation of donor slides is time-consuming and challenging for printing multiple materials or cell types. These technical limitations, along with the cost of laser sources, inhibit the generation of clinically relevant 3D constructs and the widespread use of this system.

Stereolithography is an additional nozzle-free technique in which a reservoir of photo cross-linkable material or resin is irradiated either by a laser or patterned UV light [225]. Exposure to the light source crosslinks the material, allowing for a layer-by-layer construction of thick, complex 3D structures. Stereolithographic printers make possible very rapid fabrication of complex structures with unequalled resolution of 6 μm [226]. In addition, stereographically printed structures exhibit strong interlayer bonding [219]. Due to the nature of this technique, the only method for crosslinking is photo-induced, which requires the addition of photo-initiating chemicals to initiate crosslinking [206]. Unfortunately, the most commonly used photo-initiating chemicals and UV light can also affect cell viability due to their interference with growth signaling pathways [227]. The material limitations of this technique require a viscosity of < 5 Pa/s and the ability to be photo-crosslinked, thereby considerably reducing the variety of printable materials for use in tissue engineering applications to either modified natural polymers or synthetic polymers. Furthermore, the need for a reservoir of material for printing limits the material to only one cell type, preventing the formation of complex tissues with multiple cell types or regions.

## Conclusion and perspectives

**Fig. 5:**
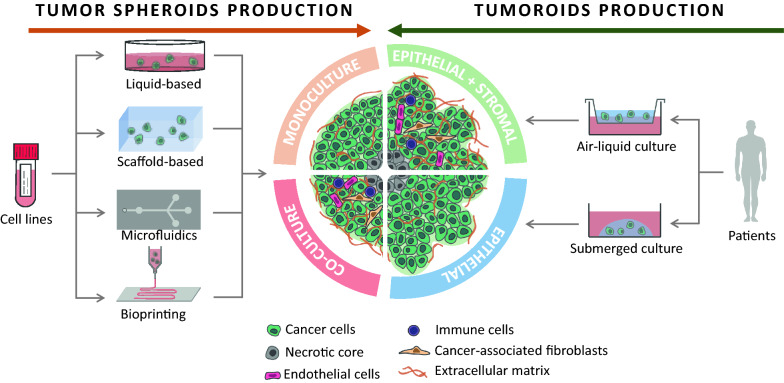
3D culture models for spheroids or tumoroids production. Tumor spheroids are often generated from cell lines, through liquid based-, scaffold based-, microfluidics or bioprinting methods. Depending on the cells added to the model, the tumor spheroid will be mainly composed of cancer cells and other cells and components of the microenvironment can be added. Tumor spheroids often show a round shape. Tumor organoids (or tumoroids) are usually generated from patient tissue samples by using two methods: (i) The submerged culture method that allows the amplification of epithelial cancer stem cells which are then able to produce ECM; (ii) The air–liquid culture method that allows the inclusion of stromal components to the tumoroids. Since tumoroids are self-organizing tissues, they will have a more complex structure than spheroids

The multiplicity of factors responsible for the different forms of cancer has encouraged scientists to use various in vitro study systems. The first and most widely used method is 2D cell culture. With 2D cell cultures, it is possible to control experiment parameters with high precision by reducing these parameters to the bare minimum. If 2D cell culture simplicity is its added value, it is a reductive model that cannot depict the complexity of cancer. 3D culture is a promising cell-based method. Here, we described and compared the advantages and limitations of the techniques that have been developed over the years to decipher the development of cancer. These techniques, which include liquid-based, scaffold-based and emerging 3D culture systems such as microfluidic platforms and bioprinting, incorporate morphological features that cannot be attained by 2D cultures and that influence the behavior of cancer cells and their microenvironment (Fig. 5).

As promising as these 3D models are, various challenges come from their use. First, the choice of the 3D culture methods depends on the scientific question raised and consequently each culture method responds to different purposes. Spheroids generated with liquid-based approaches such as the liquid overlay technique are the most straightforward way to do HTS and allow functional studies of compounds on cancer cell such viability or differentiation. However, these approaches may not consider the physical and chemical role of the microenvironment regarding drug resistance. For instance, for osteosarcoma that specifically arises in bone microenvionment, the use of mineralized scaffolds reproduce the “natural” CSCs niche and are much more adapted than soft extracellular matrix [228]. Similarly, using stationary model with or without scaffold may be inadequate to study processes that involve fluid movements such as the metastatic mechanism and in that case, microfluidic platforms would be more appropriate. Using even more complex 3D models like organoids or 3D bioprinting should allow to better mimic the diseases and to develop personal medicine programs. The use of complex 3D models may be not adapted to HTS. In addition, the increased structural complexity of 3D cultures could also complicate their analysis.

Incorporating in silico models to biological experiments may resolve the data complexity issue. Named by analogy to the words in vitro and in vivo, in silico refers to experiments performed through computer simulation. It is strongly based on results from laboratory experiments, inference, mathematical modelling and can be associated to artificial intelligence. In silico models can be multiscale, ranging from the biomolecular level to individual cell-based and systems models [229]. Beyond their capacity to provide biological insights and serve as analysis assisting tools, in silico models can also help to refine in vitro and in vivo experiments and increase the quantity and quality of data obtained in conformity with the 3Rs principles [230].

The choice of the most adapted 3D model to the question raised is dependent to the analytical processes that will be performed. Indeed, the great majority of the current analysis methods were developed for traditional 2D cell cultures, are often not adaptable to 3D culture, and require extensive validation steps [231]. Microscopic imaging of the 3D specimen will be challenging for the following reasons: (i) the physical properties of light lead to light-scattering in very thick samples, which optical sectioning and clearing techniques can not always resolve; (ii) fluorescent probes targeting specific molecules or organelles (e.g. fluorochrome-linked antibodies, DAPI, etc.) may diffuse non-homogeneously into the spheroids, with saturation of the probes on the outer layer and may be a limitation of successful imaging [232]; (iii) similarly, the poor diffusion and imaging probes to the central core of larger spheroids may be problematic [233]. Extensive preliminary setup experiments are required, since the size, the charge, or the affinity of probes to its ligand can impair the results obtained and lead to biased characterisation of spheroids [234]. 3D culture model have a much more developed ECM that can act as a barrier or a trap for the chemical, compounds and unfortunately may be associated to diffusion issue in lysis or metabolic assays [231]. However, these issues should not be seen as setbacks. Similar to 2D culture methods, increasing use of these 3D models will lead to the development of new analytic methods.

3D culture has the potential to bridge the gap between in vitro and in vivo models. By increasing the complexity of the 3D models, it is possible to approach what is observed in vivo, and still be experimenting on human cells instead of those of another species. Moreover, by adding computer modelling, it may be possible to include the 3D models in a more systemic environment. Therefore, the future of oncology research, and especially personalized medicine, will rely on the interdisciplinary collaboration of various scientific fields such as biology, medicine, physics, engineering, bioinformatics, and mathematics.

## Abbreviations

3D: Three-dimensional
ASC: Adult stem cells
CAFs: Cancer-associated fibroblasts
CAR-T: Chimeric antigen receptor T
ccRCC: Clear cell renal cell carcinoma
COs: Colon organoids
CRC: Colorectal cancer
CSCs: Cancer stem-like cells
CTCs: Circulating tumor cells
ECM: Extracellular matrix
ESC: Embryonic stem cells
ETV2: ETS variant transcription factor 2
EVs: Extracellular vesicles
FDA: Food and Drug Administration
GAGs: Glycosaminoglycans
GBOs: Glioblastoma organoids
GelMA: Gelatin methacryloyl
GEMs: Genetically engineered animal models
HA: Hyaluronic acid
HIF: Hypoxia-inducible factor
HTS: High throughput screening
iPSC: Induced pluripotent stem cells
LC–MS/MS: Liquid chromatography with tandem mass spectrometry
LEC: Laminin, entactin and type-IV collagen matrix
LGR-5: Leucine-rich repeat-containing G protein-coupled receptor 5
MALDI-MSI: Matrix-assisted laser desorption/ionization mass spectrometry imaging
MOI: Magnetic iron oxide
NSCL: Non-small lung cancer
PBMC: Peripheral blood mononuclear cells
PDMS: Polydimethylsiloxane
PEG: Polyethylene glycol
PGA: Polyglycolic acid
PGE2: Prostaglandin E2
PLA: Polylactic acid
PLC: Polycaprolactone
PLGA: Polylactic-co-glycolic acid
R-VEC: ‘Reset’ vascular endothelial cells
RCCS: Rotary cell culture system
RGD: Arginine-glycine-aspartic acid motif
RWV: Rotating wall vessel
SAPs: Self-assembling peptides
TME: Tumor microenvironment
YAP: Yes-associated protein
